# Bioinformatics research in the Asia Pacific: a 2007 update

**DOI:** 10.1186/1471-2105-9-S1-S1

**Published:** 2008-02-13

**Authors:** Shoba Ranganathan, Michael Gribskov, Tin Wee Tan

**Affiliations:** 1 Department of Chemistry and Biomolecular Sciences and Biotechnology Research Institute, Macquarie University, Sydney NSW 2109, Australia; 2 Department of Biochemistry, Yong Loo Lin School of Medicine, National University of Singapore, 8 Medical Drive, Singapore 117597; 3 Department of Biological Sciences, Purdue University, Lilly Hall of Life Sciences 915 W. State Street, West Lafayette IN 47907-2054, USA

## Abstract

We provide a 2007 update on the bioinformatics research in the Asia-Pacific from the Asia Pacific Bioinformatics Network (APBioNet), Asia's oldest bioinformatics organisation set up in 1998. From 2002, APBioNet has organized the first International Conference on Bioinformatics (InCoB) bringing together scientists working in the field of bioinformatics in the region. This year, the InCoB2007 Conference was organized as the 6^th ^annual conference of the Asia-Pacific Bioinformatics Network, on Aug. 27–30, 2007 at Hong Kong, following a series of successful events in Bangkok (Thailand), Penang (Malaysia), Auckland (New Zealand), Busan (South Korea) and New Delhi (India). Besides a scientific meeting at Hong Kong, satellite events organized are a pre-conference training workshop at Hanoi, Vietnam and a post-conference workshop at Nansha, China. This Introduction provides a brief overview of the peer-reviewed manuscripts accepted for publication in this Supplement. We have organized the papers into thematic areas, highlighting the growing contribution of research excellence from this region, to global bioinformatics endeavours.

## Introduction

The Asia-Pacific Bioinformatics Network (APBioNet, [1-3]) was established in 1998 [4] to promote the advancement of bioinformatics in the Asia Pacific region. Annual meetings initially held at the Pacific Symposium of Biocomputing (1998–2001) bore fruit in 2002 as the APBioNet executive committee members facilitated the inauguration of the International Conference on Bioinformatics in Bangkok, Thailand to promote the quality of bioinformatics research in the region. Subsequent conferences followed in Penang, Malaysia (2003); Auckland, New Zealand (2004); Busan, South Korea (2005) and New Delhi, India (2006). InCoB 2007 was held at Hong Kong [5].

APBioNet's initial efforts were focused on developing the network infrastructure with the Asia Pacific Advanced Network (APAN) [6], capable of supporting the rapid dissemination of bioinformatics databases and computational resources throughout the region. One of the services developed since 1998 was the BioMirrors initiative [7] which is currently being expanded to reach developing countries [8]. By 2000, APBioNet started to focus on bioinformatics education and training of the life science community, with active participation in e-learning initiatives such as the S* Life Science Informatics Alliance [9], to bring bioinformatics into mainstream bioscience research. Today, a critical mass of scientists in the region is now available to extend the number of conferences in bioinformatics, ranging from the Genome Informatics Workshop (GIW) [10] based mainly in Japan to the Asia Pacific Bioinformatics Conferences (APBC), InCoB [5], the International Life Science Grid Workshops (LSGRID) [11], the World Wide Workflow Grid conference (2007), and many others. In recognition of the tremendous growth of bioinformatics in the Asia Pacific, even the International Society for Computational Biology (ISCB) (MG is the immediate past President), to which APBioNet is affiliated, chose to hold one of its annual flagship ISMB conference in this region in 2003 [12]. High quality research papers from Asia Pacific researchers have started to appear in bioinformatics journals originating in the region, such as the Journal of Bioinformatics and Computational Biology (World Scientific, Singapore) [13], Applied Bioinformatics (originally from New Zealand) and Bioinformation [14].

Since 2006, when bioinformatics research in the region reached a standard, requiring international peer-reviewed high-impact factor journal publication, we have embarked on establishing international standards in bioinformatics research through this vehicle of a dedicated BMC Bioinformatics supplement [15], now in its second year. This year, we have manuscripts submitted by APBioNet members spanning several active research areas, such as the boutique database development; data and text mining; ontologies and controlled vocabularies; analyses of genome, transcriptome and protein structures; immunoinformatics; networks, pathways and systems biology, and evolution and phylogenetic analysis.

### Proceedings summary

Papers submitted to these proceedings were peer-reviewed by at least two reviewers, from the APBioNet/InCoB editorial board members and external experts as required. Of the 48 manuscripts short listed for oral presentation from the 113 submission (oral presentation acceptance rate of 46%), only 22 papers were selected (48% of orals), leading to an overall acceptance rate of 19.5% of submissions. The innovative bioinformatics research from the region is reflected in these accepted papers coauthored from Australia, China, Hong Kong, Hungary, Iran, Korea, Singapore, Taiwan, UK and USA, which fall into several general themes as described in the following sections.

### Data organization

The analysis of single nucleotide polymorphisms (SNPs) is becoming a key research area in genomics, with implications for susceptibility to diseases, prognosis of treatment regimens and for personalized healthcare. In this realm, Kim *et al. *[16] have developed the SNP@Promoter database to predict functional SNPs in putative promoter regions and transcription factor binding sites.

### Data and text mining

The difficulty in retrieving the vast amount of experimentally verified protein-protein data is addressed by a novel text-mining approach developed by Tsai *et al. *[17], while Ganapathiraju *et al. *[18] have combined latent semantic analysis with sequence features for successful prediction of transmembrane helices.

### Ontologies and controlled vocabularies

In the emerging field of lipidomics, critical to the treatment of diseases such as Alzheimer's syndrome, bacterial infections and cancer, the lack of a lipid ontology system is ably addressed by Baker *et al. *[19]. The efficient querying of the Gene Onotology resource using a Resource Description Framework is addressed by the GORouter system developed by Xu *et al. *[20]. Miotto *et al. *[21] provide a practical application of semantic technologies by annotating over 40,000 influenza A protein sequences by combining information from more than 90,000 published documents.

### Genome and transcriptome analyses

To improve the performance of gene expression arrays, Chen *et al. *[22] have developed a Unique Probe Selector web service, while Zhao and coworkers [23] describe a multivariate Bayesian model for the efficient identification of differentially expressed genes. Nagaraj *et al. *[24] have evaluated the efficacy of their semi-automatic EST analysis platform, ESTExplorer, in analysing data, and demonstrate that computational tools can be used to accelerate the process of gene discovery in EST sequencing projects.

### Structural bioinformatics

New methodologies for protein structure analysis using graph theory [25] and domain boundary prediction using an improved general regression network [26] are presented. Chelliah and Taylor [27] use functional site prediction to select correct 3D model structures. Structural modeling has been applied to the transmembrane regions of G protein-coupled receptors [28], to detect determinants in the mature peptide influencing signal peptide cleavage in signal peptidase I [29] and to discriminate between active and inactive allosteric modulator binding conformational states in metabotropic glutamate receptors [30]. Molecular dynamic simulations provide an insight into the stability of the core domain of the tumour suppressor protein, p53 [31].

### Immunoinformatics

In the global fight against disease, host-pathogen complementarity has been analysed using the AVANA system [32] for mutual information analysis, while the HotSpot Hunter [33] web service performs large-scale screening and selection of candidate immunological hotspots in pathogen proteomes.

### Networks, pathways and systems biology

Protein-protein interactions and the networks they form are essential to the fundamental understanding of how biological pathways function. Kim *et al. *[34] describe the protein interaction network in a model cyanobacterium while Wang *et al. *[35] have developed a novel computational approach to integrate motif information and gene expression data for regulatory network reconstruction.

### Evolution and phylogenetic analyses

The evolution of genes, especially in response to pathogenic attack, is a complex process. Kong and Ranganathan [36] have combined gene, protein, domain, motif and evolutionary analyses of the potato inhibitor II family, to understand the strategies developed by *Solanaceae *plants for defense against pathogenic attacks. The informatics challenges posed by large scale phylogenetic analysis have prompted Singh *et al*. [37] to develop Quascade, a distributed computing platform, for phyloinformatics, for the rapid analysis of viral sequences, and for monitoring pathogen evolution.

## Conclusion

As exemplified in this special issue, and by a wide range of publications from other conferences mentioned in this editorial, it is clear that Asia Pacific bioinformatics research is thriving. To continue this trend, it is imperative that bioinformatics education becomes entrenched in the curriculum of our institutions of higher learning. In this regard, the efforts of the Workshop on Education in Bioinformatics (WEB) [38] (as initiated by one of the co-authors, SR) and other training and policy meetings coordinated and facilitated by APBioNet, such the ASEAN-India Bioinformatics Workshop series, the ASEAN-China Bioinformatics Workshop series and the East Asia Bioinformation Network meetings (coordinated by one of the co-authors, TTW) [39], will play key roles in each Asia Pacific country.

As a new generation of life science researchers trained to practise *in silico *biology in addition to *in vitro *and *in vivo *life science emerges, the need for resources will increase. The network and computational infrastructure to support such new demands will require a new biocyberinfrastructure to be provisioned in each locality, whether it is distributed computing facilities such as grid computing and cloud computing, or in-campus centralized resources.

Growth in research quality, education excellence and resource provisioning will need to take place hand-in-hand. Organisations such as APBioNet and the recently formed Asian Association for Societies in Bioinformatics (AASBi) [40] are already formulating their policies to foster such growth. Collaborations with projects such as the TEIN2 initiative [41] to build broadband regional network connectivity and with international organisations such as ISCB will accelerate the sustained growth of bioinformatics in the Asia Pacific.

To this end, bioinformatics champions in each country and in each institution are called to come forward to lead this growth and show the way forward. Equally, each individual scientist is urged to adopt and promote techniques in computational biology and bioinformatics as a routine part of their arsenal of tools to be brought to bear on 21^st ^century biology.

## Competing interests

The authors declare that they have no competing interests.

## References

[B1] The Asia-Pacific Bioinformatics Network. http://apbionet.org.

[B2] Miyano S, Ranganathan S (2001). The Asia-Pacific Regional Perspective on Bioinformatics. IEEE Intelligent Systems.

[B3] Ranganathan S, Subbiah S, Tan TW (2002). APBioNet: the Asia-Pacific regional consortium for bioinformatics. Appl Bioinformatics.

[B4] Sugawara H, Miyazaki S (1998). Towards the Asia-Pacific Bioinformatics Network. Pac Symp Biocomput.

[B5] The Sixth International Conference on Bioinformatics (InCoB2007): HKUST, Hong Kong; Hanoi, Vietnam and Nansha, PR China. http://incob.apbionet.org/.

[B6] Asia Pacific Bioinformatics Network (APAN). http://www.apan.net.

[B7] Gilbert D, Ugawa Y, Buchhorn M, Tan TW, Mizushima A, Kim H, Chon K, Weon S, Ma J, Ichiyanagi Y, Liou DM, Keretho S, Napis S (2004). Bio-Mirror project for public bio-data distribution. Bioinformatics.

[B8] Sangket U, Phongdara A, Chotigeat W, Nathan D, Kim W-Y, Bhak J, Ngamphiw C, Tongsima S, Khan AM, Lin HH, Tan TW (2007). Automatic synchronization and distribution of biological databases and software over low-bandwidth networks among developing countries. Bioinformatics.

[B9] Lim YP, Höög JO, Gardner P, Ranganathan S, Andersson S, Subbiah S, Tan TW, Hide W, Weiss AS (2003). The S-Star Trial Bioinformatics Course: An On-Line Learning Success. Biochem Mol Biol Edu.

[B10] Genome Informatics Workshop. http://giw.ims.u-tokyo.ac.jp/giw/index.html.

[B11] International Life Science Grid Workshop (LSGRID). http://www.lsgrid.org/.

[B12] 11th International Conference on Intelligent Systems for Molecular Biology (ISMB), Brisbane, Australia, June 29 – July 3, 2003. http://www.iscb.org/ismb2003/.

[B13] Journal of Bioinformatics and Computational Biology (JBCB). http://www.worldscinet.com/jbcb/jbcb.shtml.

[B14] Bioinformation. http://bioinformation.net.

[B15] Ranganathan S, Tammi M, Gribskov M, Tan TW (2006). Establishing bioinformatics research in the Asia Pacific. BMC Bioinformatics.

[B16] Kim BC, Kim WY, Park D, Chung WH, Shin KS, Bhak J (2008). SNP@Promoter: a database of human SNPs (Single Nucleotide Polymorphisms) within the putative promoter regions. BMC Bioinformatics.

[B17] Tsai RTH, Hung HC, Dai HJ, Lin YW, Hsu WL (2008). Exploiting likely-positive and unlabeled data to improve the identification of protein-protein interaction articles. BMC Bioinformatics.

[B18] Ganapathiraju M, Balakrishnan N, Reddy R, Klein-Seetharaman J (2008). Transmembrane helix prediction using amino acid property features and latent semantic analysis. BMC Bioinformatics.

[B19] Baker CJO, Kanagasabai R, Ang WT, Veeramani A, Low HS, Wenk MR (2008). Towards ontology-driven navigation of the lipid bibliosphere. BMC Bioinformatics.

[B20] Xu Q, Shi Y, Lu Q, Zhang G, Luo Q, Li Y (2008). GORouter: an RDF model for providing semantic query and inference services for gene ontology and its associations. BMC Bioinformatics.

[B21] Miotto O, Tan TW, Brusic V (2008). Rule-based Knowledge Aggregation for Large-Scale Protein Sequence Analysis of Influenza A Viruses. BMC Bioinformatics.

[B22] Chen SH, Lo CZ, Tsai MC, Hsiung CA, Lin CY (2008). The unique probe selector: a comprehensive web service for probe design and oligonucleotide arrays. BMC Bioinformatics.

[B23] Zhao H, Chan KL, Cheng LM, Yan H (2008). Multivariate hierarchical Bayesian model for differential gene expression analysis in microarray experiments. BMC Bioinformatics.

[B24] Nagaraj SH, Gasser RB, Nisbet AJ, Ranganathan S (2008). *In silico *analysis of expressed sequence tags from *Trichostrongylus vitrinus *(Nematoda): comparison of the automated ESTExplorer workflow platform with conventional database searches. BMC Bioinformatics.

[B25] Ördög R, Szabadka Z, Grolmusz V (2008). Analyzing the simplicial decomposition of spatial protein structures. BMC Bioinformatics.

[B26] Yoo PD, Sikder AR, Zhou BB, Zomaya AY (2008). Improved general regression network for protein domain boundary prediction. BMC Bioinformatics.

[B27] Chelliah V, Taylor WR (2008). Functional site prediction selects correct protein models. BMC Bioinformatics.

[B28] Dastmalchi S, Church WB, Morris MB (2008). Modelling the structures of G protein-coupled receptors aided by three-dimensional validation. BMC Bioinformatics.

[B29] Choo KH, Tong JC, Ranganathan S (2008). Modeling *Escherichia coli *signal peptidase complex with bound substrate: determinants in the mature peptide influ-encing signal peptide cleavage. BMC Bioinformatics.

[B30] Yanamala N, Tirupula KC, Klein-Seetharaman J (2008). Preferential binding of allosteric modulators to active and inactive conformational states of metabotropic glutamate receptors. BMC Bioinformatics.

[B31] Madhumalar A, Smith DJ, Verma C (2008). Stability of the core domain of p53: insights from computer simulations. BMC Bioinformatics.

[B32] Miotto O, Heiny AT, Tan TW, August JT, Brusic V (2008). Identification of human-to-human transmissibility factors in PB2 proteins of influenza A by large-scale mutual information analysis. BMC Bioinformatics.

[B33] Zhang GL, Khan AM, Srinivasan KN, Heiny AT, Lee KX, Kwoh CK, August JT, Brusic V (2008). Hotspot Hunter: a computational system for large-scale screening and selection of candidate immunological hotspots in pathogen proteomes. BMC Bioinformatics.

[B34] Kim WY, Kang S, Kim BC, Oh J, Cho S, Bhak J, Choi JS (2008). SynechoNET integrated protein-protein interaction database of a model cyanobacterium *Synechocystis *sp. PCC 6803. BMC Bioinformatics.

[B35] Wang C, Xuan J, Chen L, Zhao P, Wang Y, Clarke R, Hoffman E (2008). Motif-directed network component analysis for regulatory network inference. BMC Bioinformatics.

[B36] Kong L, Ranganathan S (2008). Tandem duplication, circular permutation, molecular adaptation: how *Solanaceae *resist pests *via *inhibitors. BMC Bioinformatics.

[B37] Singh DT, Trehan R, Schmidt B, Bretschneider T (2008). Comparative phyloinformatics of virus genes at micro and macro levels in a distributed computing environment. BMC Bioinformatics.

[B38] Ranganathan S (2005). Bioinformatics education – perspectives and challenges. PLoS Comput Biol.

[B39] East Asia Bioinformation Network (EABN). http://eabn.apbionet.org.

[B40] The Association of Asian Societies for Bioinformatics. http://www.aasbi.org/.

[B41] Trans-Eurasia Information Network (TEIN). http://www.tein2.net.

